# Differential auditory-oculomotor interactions in patients with right vs. left sided subjective tinnitus: a saccade study

**DOI:** 10.3389/fnhum.2013.00047

**Published:** 2013-02-26

**Authors:** Alexandre Lang, Marine Vernet, Qing Yang, Christophe Orssaud, Alain Londero, Zoï Kapoula

**Affiliations:** ^1^CNRS CESEM - UMR 8194, Université Paris DescartesParis, France; ^2^Service d'Ophtalmologie, Hôpital Européen Georges PompidouParis, France; ^3^Service d'ORL et de Chirurgie Cervico-Faciale, Hôpital Européen Georges PompidouParis, France

**Keywords:** asymmetry, cross-modal, interactions, tinnitus, visually guided saccades

## Abstract

Subjective tinnitus (ST) is a frequent but poorly understood medical condition. Recent studies demonstrated abnormalities in several types of eye movements (smooth pursuit, optokinetic nystagmus, fixation, and vergence) in ST patients. The present study investigates horizontal and vertical saccades in patients with tinnitus lateralized predominantly to the left or to the right side. Compared to left sided ST, tinnitus perceived on the right side impaired almost all the parameters of saccades (latency, amplitude, velocity, etc.) and noticeably the upward saccades. Relative to controls, saccades from both groups were more dysmetric and were characterized by increased saccade disconjugacy (i.e., poor binocular coordination). Although the precise mechanisms linking ST and saccadic control remain unexplained, these data suggest that ST can lead to detrimental auditory, visuomotor, and perhaps vestibular interactions.

## Introduction

Subjective tinnitus (ST) is an auditory percept perceived in the absence of any external or internal auditory stimulus. ST is experienced by around 10% of the population and can strongly impair the quality of life (Holmes and Padgham, 2011). Pathophysiology of ST remains poorly understood despite extensive animal and human research aiming at deciphering the neural correlates of such illusory percept to promote effective treatments (Elgoyhen et al., 2012). However, there is strong evidence suggesting that an auditory peripheral insult and related deafferentation could trigger complex neuroplastic subcortical and cortical maladaptive changes involving auditory and non-auditory brain areas (Roberts et al., 2010).

Obviously the causes of this multifaceted audiologic symptom are multifactorial even if epidemiologic data have shown that the main medical condition related to it is hearing loss independently of its cause (Nicolas-Puel et al., 2006; Mazurek et al., 2010). Peripheral auditory impairment can indeed be considered as a trigger. But to explain both tinnitus persistence and tinnitus induced intolerance, different pathophysiological models (for a review, see Kaltenbach, 2011) highlight the crucial role of sub-cortical and cortical plasticity, resulting from the maladaptive efforts of auditory and non-auditory pathways to compensate such deficit (Yang et al., 2011).

Different intriguing tinnitus clinical patterns show up this central involvement. Among them, somatic modulation of tinnitus features (intensity, frequency) with specific movements (jaw protrusion, head rotation, muscular contraction, etc.) constitutes a specific sub-type of tinnitus (Levine, 1999; Abel and Levine, 2004). Interestingly, lateral gaze has also been shown to interact with ST percept (Coad et al., 2001). These somatic modulations of ST are putatively supported by the cross-modal wiring and talking between somatosensory and auditory pathways. This has been demonstrated at a sub cortical level (dorsal cochlear nuclei, inferior colliculi) via a trigeminal modulation of auditory function in a behavioral animal model of ST induced by an auditory damage (Shore et al., 2007). Neuronal pathways, by which the somatic afferences interact with the central auditory pathways, are not ascertained. But animal data (Shore et al., 2003) support the implication of the dorsal cochlear nucleus, via a trigeminal input, as an important hub for auditory and somatic bimodal interaction especially after cochlear damage (Dehmel et al., 2008). In humans, jaw protrusion modulation of tinnitus assessed with functional imaging (fMRI) also showed increased activation in cochlear nuclei and inferior colliculi but decreased cerebellar activation when compared to controls (Lanting et al., 2010). PET data on gaze evoked tinnitus support the hypothesis of the emergence of abnormal links between brainstem systems controlling eye movements and auditory structures (Lockwood et al., 2011). Moreover an interaction of somatosensory stimulation (TENS) with tinnitus loudness has been reported in tinnitus patients (Vanneste et al., 2011a) and has been interpreted as a clue for the activation of the non-specific extralemniscal pathways ending into parietal cortices (Møller, 2007). Neural plasticity induced by tinnitus could then interact at many different levels with neural circuitries involved in saccade programming and execution.

Indeed, cross-modal interaction of auditory, visual, and somatosensory inputs is obvious because in our dynamic and changing environment we constantly shift our gaze and move our body toward detected relevant auditory objects. We also need to be able to compensate one sense with another when one modality fails i.e., vision in darkness or audition in a noisy atmosphere (Maier and Groh, 2009). Of particular interest in the perspective of the present paper is the question whether there is a different clinical pattern between left sided and right sided tinnitus. Actually, clinical evidence indicates that in many cases tinnitus is lateralized, at least it is perceived predominantly to the left or to the right of the median sagittal plane and that tinnitus laterality is related to different clinical features (Mazurek et al., 2010).

Interestingly, recent studies demonstrated that ST could impair oculomotor performance both during fixation, smooth pursuit, and optokinetic nystagmus movements (Coad et al., 2001; Jozefowicz-Korczynska and Pajor, 2002; Mezzalira et al., 2007; Kapoula et al., 2010; Lockwood et al., 2011), and also in vergence movements (Yang et al., 2010). To our knowledge, quantitative studies of saccades in ST patients have not yet been done, leaving alone the possible differences between right and left sided tinnitus. Saccades are among the most robust eye movements. Yet fine analysis of their spatial temporal properties might be affected by tinnitus. This study examines the parameters of ocular saccades toward four directions (left, right, up, and down) in patients suffering from lateralized (predominantly left or predominantly right) chronic ST. Many multiple parameters of the saccades were examined related to cortical-subcortical brainstem functions. These parameters concern the preparation of the saccade (latency, drift of the eyes before the start of the saccade), its execution (amplitude, velocity, saccade disconjugacy), and the immediate period after its completion (drift after the saccade). The study focuses on the comparison of left vs. right tinnitus patients aiming to assess relative differences and the degree of optimal behavior of the saccade system that could be maintained despite the tinnitus. A comparison will also be made with data from healthy subjects from two other studies from our group (Vernet et al., 2008a,b) using similar experimental setups.

## Materials and methods

### Ethics statement

The eye movement investigation adhered to the tenets of the Declaration of Helsinki and was approved by the local ethics committee for human experimentation, CPP Ile de France II (No: 07035, Hospital Necker in Paris). Written consent approved by the committee was obtained from all subjects after the nature of the examination had been explained.

### Subjects and clinical data

A total of 20 patients (47.5 ± 12.6 years) volunteered to participate in this experiment. All patients attended a tertiary care tinnitus clinic at European Hospital Georges Pompidou in Paris; tinnitus perception being stated as their main medical complaint. They were selected for this study because of their ability to modulate their tinnitus by somatic stimuli (movements, muscle pressure). Complete otologic and neurologic testing was performed on each subject (audiometry, tympanometry, stapedial reflexes, auditory evoked potential, and/or MRI). All patients suffered from tinnitus for at least 1 year (6 years on average). Tinnitus was predominantly left sided in 12 patients and predominantly right sided in eight patients. Table 1 summarizes the characteristics of both populations.

**Table 1:** **Clinical characteristics of tinnitus patients**.

**TSL subjects**	**TSR subjects**
*N* = 12 (6 males, 6 females)	*N* = 8 (5 males, 3 females)
Mean age = 44 ± 12 years	Mean age = 53 ± 12 years
Duration = 6 ± 8 years (from 1 to 30)	Duration = 6 ± 7 years (from 1 to 20)
Tinnitus severity^a^ (max = 16) = 10 ± 3	Tinnitus severity^a^ (max = 16) = 11 ± 2
Pathological condition: otological (*n* = 2), neuro-muscular (*n* = 4), idiopathic (*n* = 2)	Pathological condition: otological (*n* = 3), neuro-muscular (*n* = 2), idiopathic (*n* = 1)
Stress: *n* = 8	Stress: *n* = 3

a Tinnitus severity was evaluated with the Subjective Tinnitus Severity Scale (Halford and Anderson, 1991; Meric and Chery-Croze, 1996); Score > 8 indicates moderate tinnitus; Score > 12 indicates severe tinnitus.

Tinnitus was modulated by jaw movements in 13 patients, head movements in seven patients, muscular pressure in eight patients, eye movements in three patients, and global muscular effort in two patients. One condition elicited tinnitus modulation in 10 patients, two conditions in seven patients and three in the remaining three patients. The pathological conditions being present at the onset of tinnitus were either otological (right otitis media, left otosclerosis surgery, bilateral noise induced hearing loss, sensorineural hearing loss) or neuro-muscular (acoustic neuroma, right meningomia, meningitis, left head trauma with TMJ luxation, cervicalgia, abdominal, or orthopedic surgery). The causal link between these medical conditions and tinnitus is only putative. Unusually stressful circumstances were indicated by 11 patients; these patients had a mean score higher than 100 on the Holmes and Rahe life events related stress questionnaire (Miller and Rahe, 1997). Even if the stress is not direct cause of tinnitus it can be considered as a trigger of tinnitus intrusiveness (Andersson and Westin, 2008; Hesser and Andersson, 2009). Tinnitus was considered as idiopathic in the remaining three patients, i.e., no specific condition was associated with the onset of tinnitus. There was no difference between right sided tinnitus patients (4/8, i.e., 50%) and left sided tinnitus patients (6/12, i.e., 50%) regarding the presence of an organic disorder linked to tinnitus onset.

Standard audiometric thresholds (0.250, 0.500, 1, 2, 4, 6, 8 kHz) were normal in seven patients (audiometric threshold >20 db on each tested frequency) and demonstrated high frequency sensorineural hearing loss in nine patients, middle frequency sensory neural hearing loss in two patients, and total unilateral cophosis in two patients (acoustic neuroma patients). All patients had stable hearing levels at the time of testing with no recent impairment. This is in line with previous reports of the high prevalence of hearing impairment in tinnitus (Nicolas-Puel et al., 2002). None of them had acute clinical vestibular dysfunction at the time of testing as attested to by the absence of vertigo or dizziness or spontaneous nystagmus.

In summary, the pathophysiological mechanisms underlying the tinnitus percept probably vary among patients. Yet, this group of subjects is homogenous because of their spontaneously modulated tinnitus following different types of movements.

### Visual display

**Figure 1:**
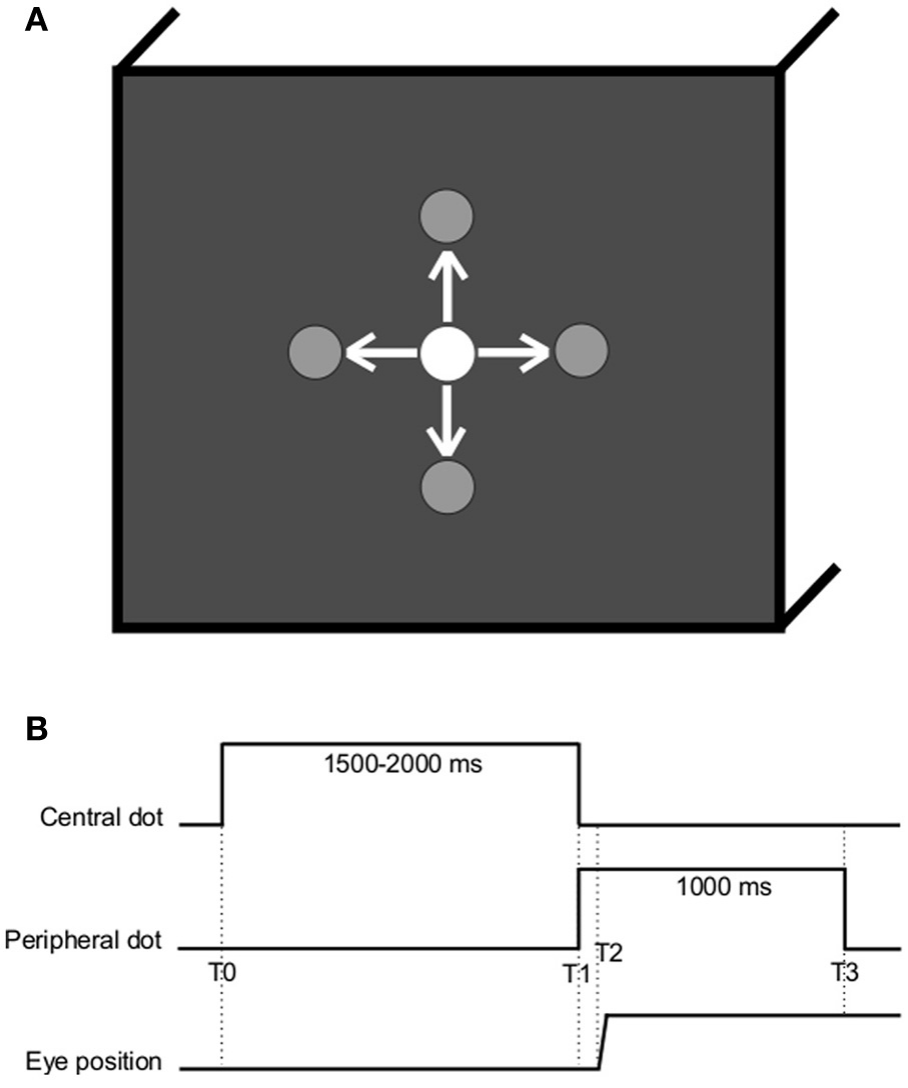
**(A)** Spatial arrangement. The white dot is here represented at the center but could be located at one of the other peripheral locations (gray), at ±10° horizontally or vertically. Arrows represent the possible saccade stimulation. **(B)** Temporal arrangement (T0, central dot turns on; T1, central dot cuts off and peripheral dot turns on; T2–T1, latency of ocular response; T3, peripheral dot cuts off).

The stimuli consisted of a white dot that was displayed at different locations on a computer screen filled with a black background as in Figure 1. Subjects were comfortably seated in an adapted chair with a chin rest to stabilize the head at about 57 cm from the computer screen.

### Eye movement recording

Horizontal and vertical eye movements were recorded binocularly with the EyeLink II device. Each channel was sampled at 250 Hz. The spatial resolution of the system was 0.025° of visual angle. Before the eye movement task, the subject made a sequence of saccades of 10° between the white dots, in the four directions; each dot was turned on for 1000 ms (a period of time allowing accurate and stable fixation). The calibration factors for each eye were extracted from these recordings; a linear function was used to fit calibration data.

### Oculomotor tasks

Subjects were instructed to gaze at the white dot as accurately as possible. The dot was successively displayed at five possible locations: center of the screen, 10° left, 10° right, 10° up, or 10° down (see Figure 1). The task involved saccade eye movements to the four directions (left, right, up, or down) from the center in every instance. Each subject was randomly presented with 10 of each type of saccades. For each of the 40 saccades, the initial central dot was turned on for a random period varying between 1500 and 2000 ms to reduce anticipation; at the immediate end of this period, the next peripheral dot was simultaneously turned on for 1000 ms (see Figure 1). Two trials were separated with a 1-s blank screen.

### Data analysis

From the two individual calibrated eyes position signals (LE: left eye; RE: right eye), we derived the conjugate (version) signal (RE/2 + LE/2) and the disconjugate (vergence) signal (LE-RE). The eye velocity of the conjugate signal was computed using a symmetrical two-point diffentiator after low-pass filtering with a Gaussian FIR filter with a cut-off frequency of 33 Hz. The start and the end of the saccade were defined as the time point when the velocity of the conjugate signal exceeded or dropped below 15°/s. For the vergence signal, the start and the end of the movement were defined as the time point when the velocity exceeded or dropped below 5°/s. These criteria are standard (Takagi et al., 1995; Yang et al., 2002). For both saccade and vergence, the process was performed automatically by the computer, and the verification was made by visual inspection of the individual eye position and velocity traces.

On the version signal, we measured for each saccade the latency, i.e., the time between the target onset (0 ms) and the start of the saccade, the amplitude, the mean velocity (amplitude/saccade duration), the drift before the saccade, i.e., the fluctuation of amplitude during the latency, the drift after the saccade, i.e., during the first 80 ms after the end of the saccade. These pre- and post-saccadic drifts were the absolute eye movements in the saccade dimension (i.e., horizontal for leftward or downward saccades and vertical for upward and downward saccades). On the vergence signal, we measured for each saccade the disconjugacy (horizontal disconjugacy for horizontal saccades, vertical disconjugacy for vertical saccades). Eye movements in the wrong direction, with latency shorter than 80 ms (anticipation) or longer than 800 ms, or contaminated by blinks were rejected.

The data on three parameters (fixation drift before the start of the saccade, fixation drift after the saccade and saccade disconjugacy) were not normally distributed; however, these data fit normal distribution after log transformation. Based on the transformed (fixation drift before and after saccades, disconjugacy) or raw (latency, amplitude, mean velocity) data, a Two-Way analysis of variance (ANOVA) was performed on individual mean values of each parameters with the between subjects factor Tinnitus Side [Tinnitus Side Left (TSL), Tinnitus Side Right (TSR)], and the within subjects factor Saccade Direction (L, Left; R, Right; U, Up; D, Down). *Post-hoc* comparisons were done with the Tukey's Honestly Significant Difference (HSD) test. The significance level was set at *p* < 0.05.

## Results

This section presents the results of ANOVA, evaluating the effects of the Tinnitus Side conditions and Saccade Direction conditions on each of the following parameters: latency and fixation drift before saccades (preparation of the saccade), amplitude and mean velocity (execution of the saccade), fixation drift after saccades, and saccade disconjugacy (binocular coordination).

### Latency

**Figure 2:**
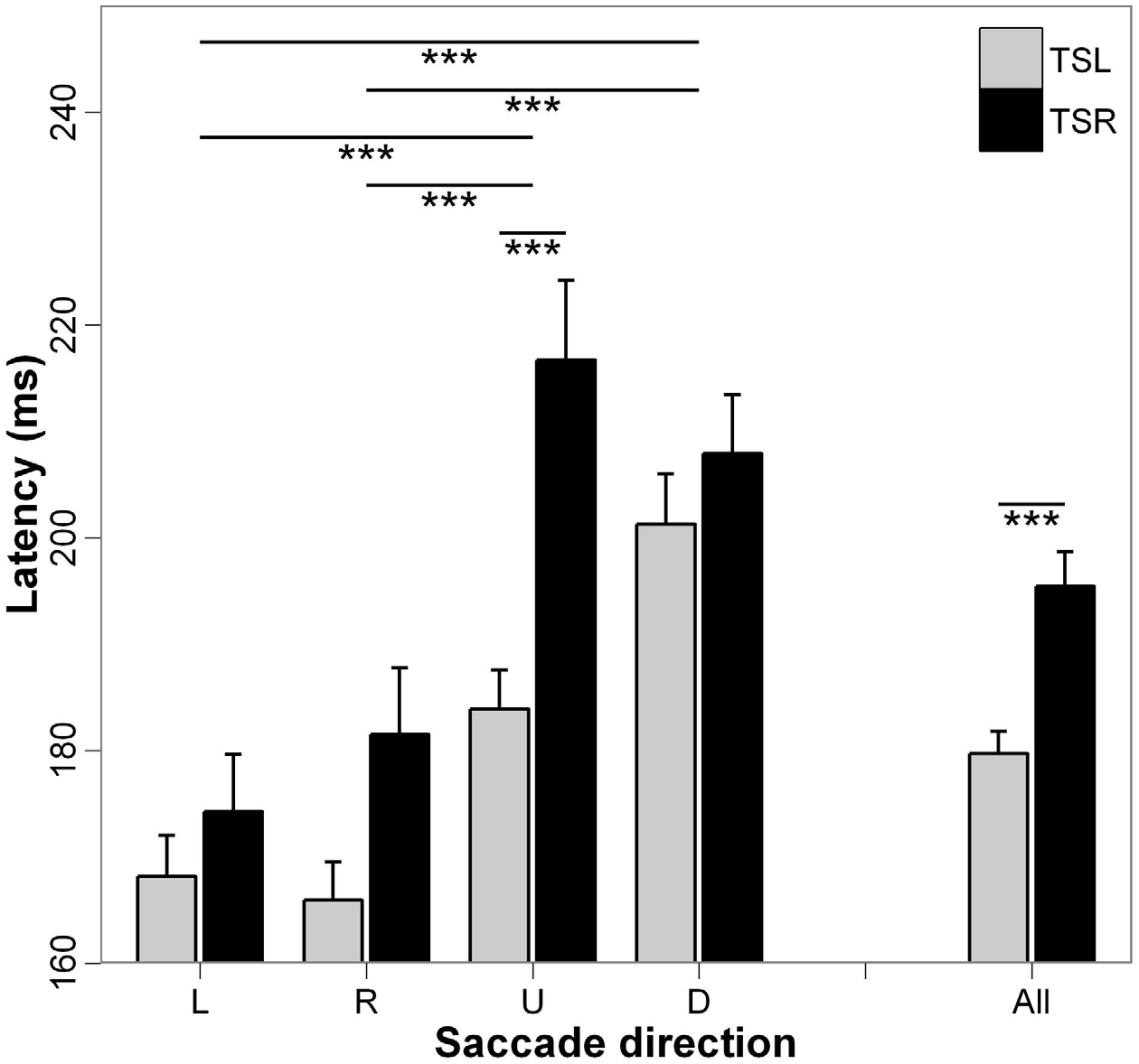
**Mean values of latency for each saccade direction (L, leftward; R, rightward; U, upward; D, downward) and tinnitus side (TSL, tinnitus side left; TSR, tinnitus side right).** Error bars represent the standard error. The symbol ^*^ indicates a statistically significant effect (^*^p < 0.05; ^**^p < 0.01; ^***^p < 0.001).

Figure 2 presents the mean latencies and standard errors for each direction of the saccades and side of the tinnitus. ANOVA applied on the mean values of latency shows statistically significant main effect of the tinnitus side [*F*_(1, 18)_ = 17.49; *p* < 0.001], i.e., higher latencies for subjects with right than those with left tinnitus, and a significant main effect of the direction of the saccades [*F*_(3, 16)_ = 22.24; *p* < 0.001], i.e., upward and downward saccades have longer latencies than leftward and rightward saccades (for each paired comparisons, *p* < 0.001; except for leftward vs. rightward saccades, *p* = 0.990, and upward vs. downward saccades, *p* = 0.504). Finally, the interaction effect between tinnitus side and saccades direction is also significant [*F*_(3, 16)_ = 2.89; *p* < 0.05), i.e., upward saccades have significantly longer latencies for right side tinnitus than for left side tinnitus (*p* < 0.001; others paired comparisons are n.s.).

### Absolute fixation drift prior to saccade start

**Figure 3:**
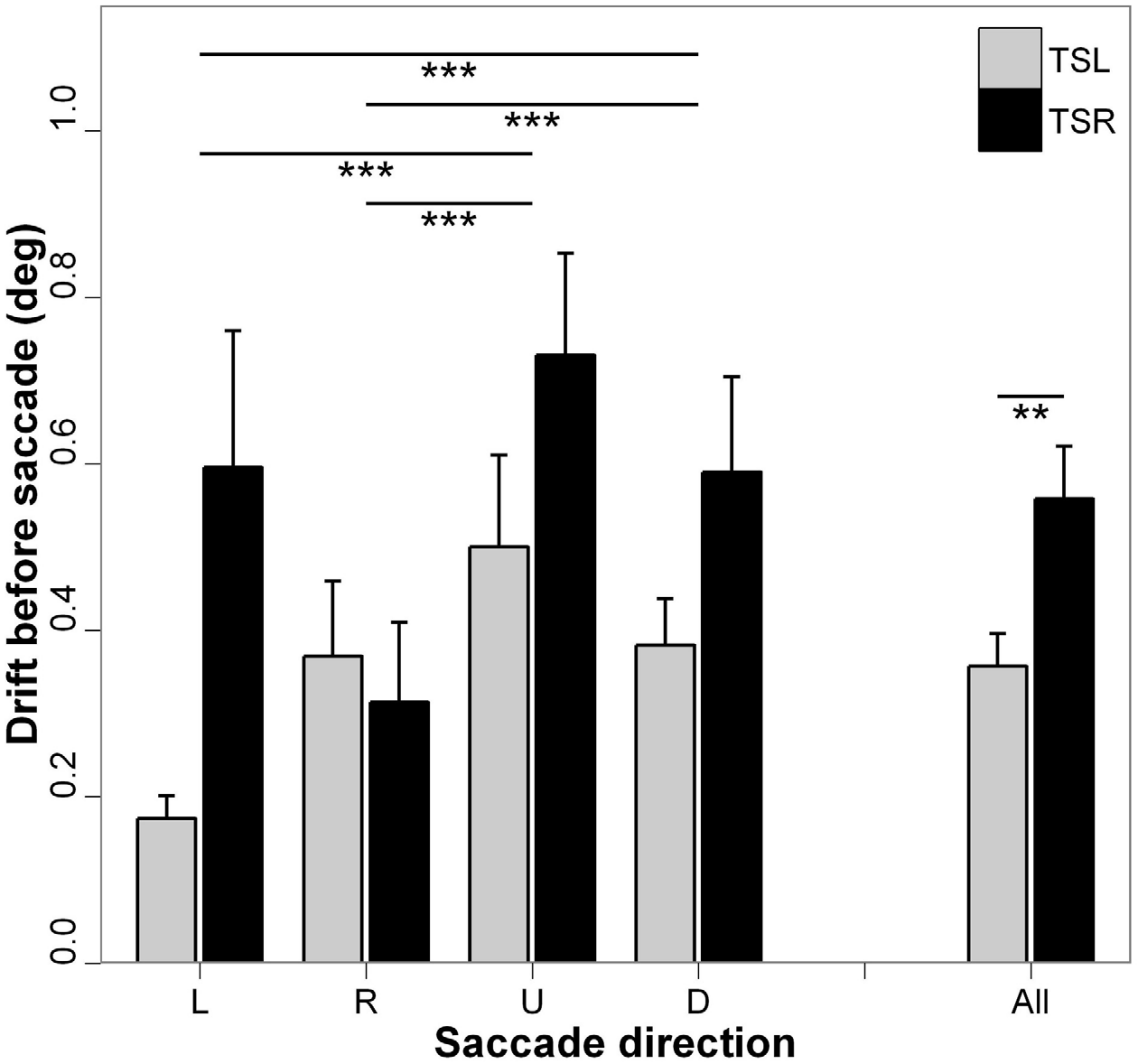
**Absolute mean values of fixation drift before saccade for each saccades direction (L, leftward; R, rightward; U, upward; D, downward) and tinnitus side (TSL, tinnitus side left; TSR, tinnitus side right).** Error bars represent the standard error. The symbol ^*^ indicates a statistically significant effect (^*^p < 0.05; ^**^p < 0.01; ^***^p < 0.001).

Figure 3 presents the absolute mean fixation drift before saccades start and standard errors for each direction of the saccades and side of the tinnitus. ANOVA shows statistically significant main effect of the tinnitus side [*F*_(1, 18)_ = 8.36; *p* < 0.01], i.e., subjects with right tinnitus drifted more from the fixated target before saccades than subjects with left tinnitus, and a significant main effect of the direction of the saccades [*F*_(3, 16)_ = 17.84; *p* < 0.001], i.e., vertical drift before a saccade to a vertical saccade target is higher than horizontal drift toward a horizontal saccade target (for each paired comparisons, *p* < 0.001; except for leftward vs. rightward saccades, *p* = 0.990, and upward vs. downward saccades, *p* = 0.792). There is no interaction effect between tinnitus side and saccades direction [*F*_(3, 16)_ = 1.61; *p* = 0.186].

### Amplitude

**Figure 4:**
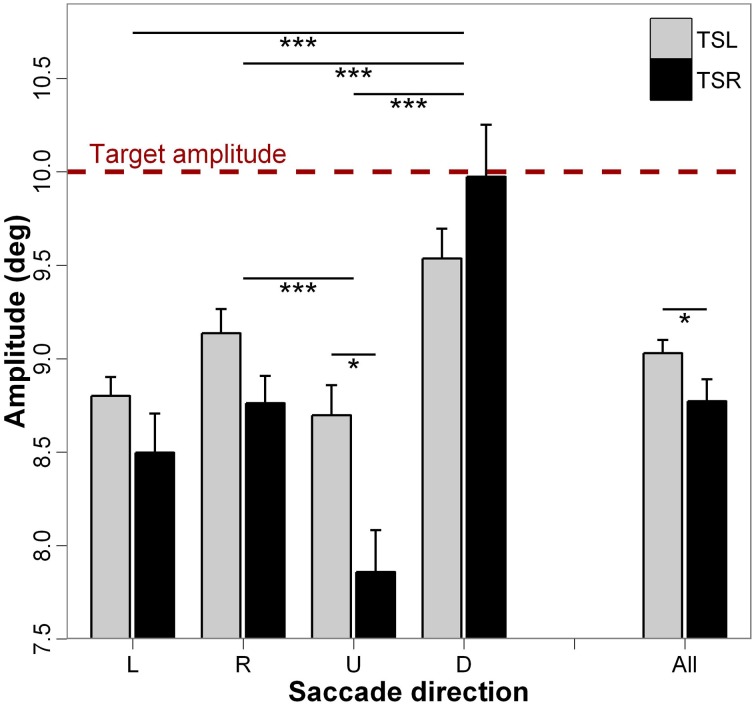
**Mean values of amplitude for each saccade direction (L, leftward; R, rightward; U, upward; D, downward) and tinnitus side (TSL, tinnitus side left; TSR, tinnitus side right).** The dashed line represents the target amplitude. Error bars represent the standard error. The symbol ^*^ indicates a statistically significant effect (^*^p < 0.05; ^**^p < 0.01; ^***^p < 0.001).

Figure 4 presents the mean amplitude of saccades and standard errors for each direction of the saccades and side of the tinnitus. ANOVA shows statistically significant main effect of the tinnitus side [*F*_(1, 18)_ = 4.49; *p* < 0.05], i.e., subjects with right tinnitus perform more hypometric amplitudes than those with left tinnitus, and significant main effect of the direction of the saccades [*F*_(3, 16)_ = 20.13; *p* < 0.001); i.e., downward saccades are less hypometric than upward, leftward, and rightward saccades (for each paired comparisons, *p* < 0.001; except for leftward vs. rightward saccades, *p* = 0.327, and upward vs. leftward saccades, *p* = 0.283). The interaction effect between tinnitus side and saccades direction is significant (*F*_(3, 16)_ = 4.04; *p* < 0.01), i.e., for upward saccades, hypometria is wider for right tinnitus than for left tinnitus side (*p* < 0.05; others paired comparisons are n.s.). It could be argued that the greater undershooting of saccade amplitude in TSR may be partly due to the greater average drift before the saccade. This appeared to be not the case because we calculated that only 49.4% of pre-saccadic drifts and of saccades were made in the same direction; in 50.6% of cases, drifts before saccades were made in the opposite direction.

### Mean velocity

**Figure 5:**
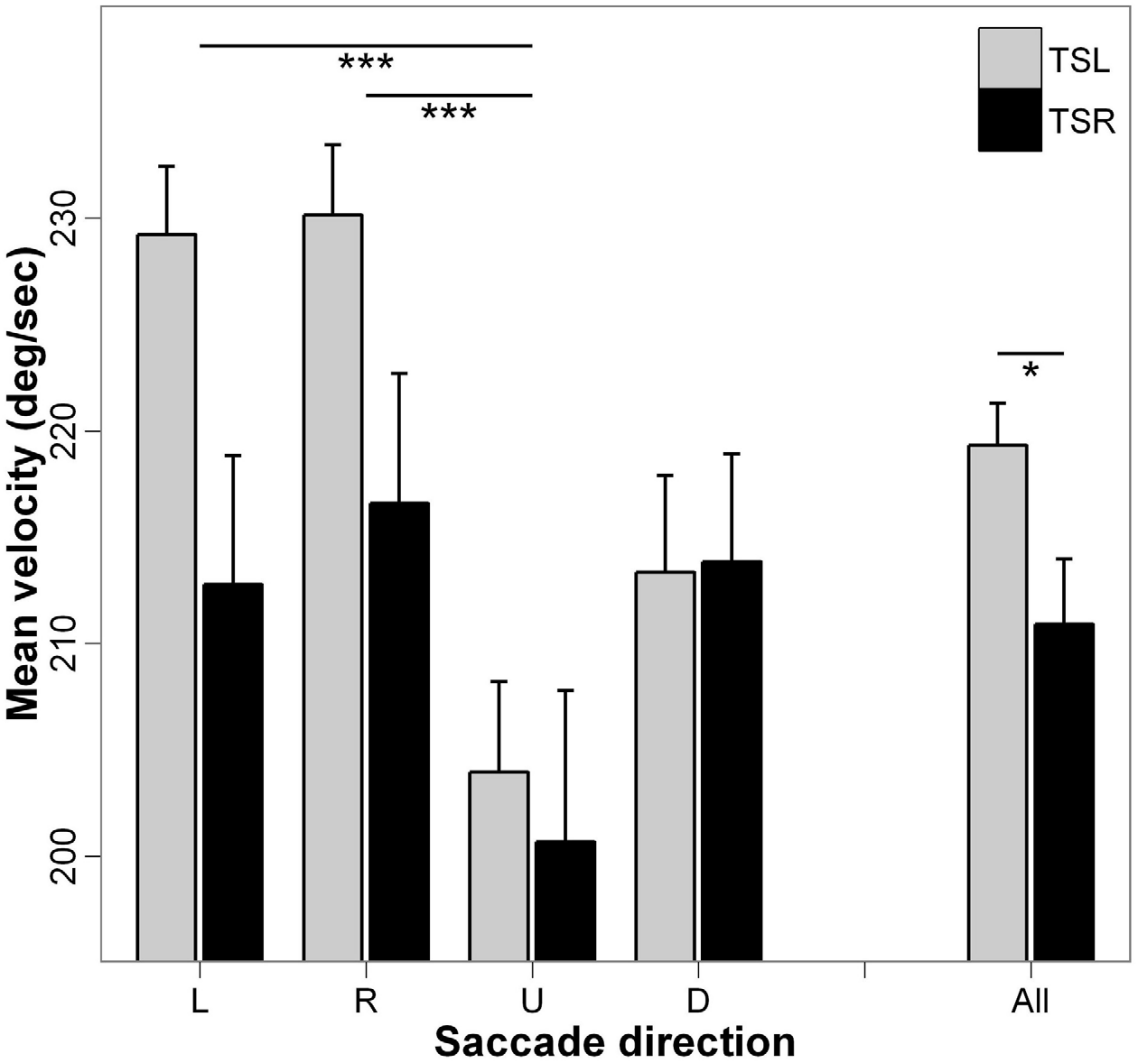
**Mean values of mean velocity for each saccade direction (L, leftward; R, rightward; U, upward; D, downward) and tinnitus side (TSL, tinnitus side left; TSR, tinnitus side right).** Error bars represent the standard error. The symbol ^*^ indicates a statistically significant effect (^*^p < 0.05; ^**^p < 0.01; ^***^p < 0.001).

Figure 5 presents the mean velocity and standard errors for each direction of the saccades and side of the tinnitus. ANOVA indicates statistically significant main effect of the tinnitus side [*F*_(1, 18)_ = 5.10; *p* < 0.05], i.e., subjects with right tinnitus performed slower saccades than those with left tinnitus, and a significant main effect of the direction of the saccades [*F*_(3, 16)_ = 8.17; *p* < 0.001], i.e., mean velocity of upward saccades is slower than that of rightward and of leftward saccades (*p* < 0.001; others paired comparisons are n.s.). There is no interaction effect between tinnitus side and saccades direction [*F*_(3, 16)_ = 1.22; *p* = 0.30].

### Absolute fixation drift after the saccade

**Figure 6:**
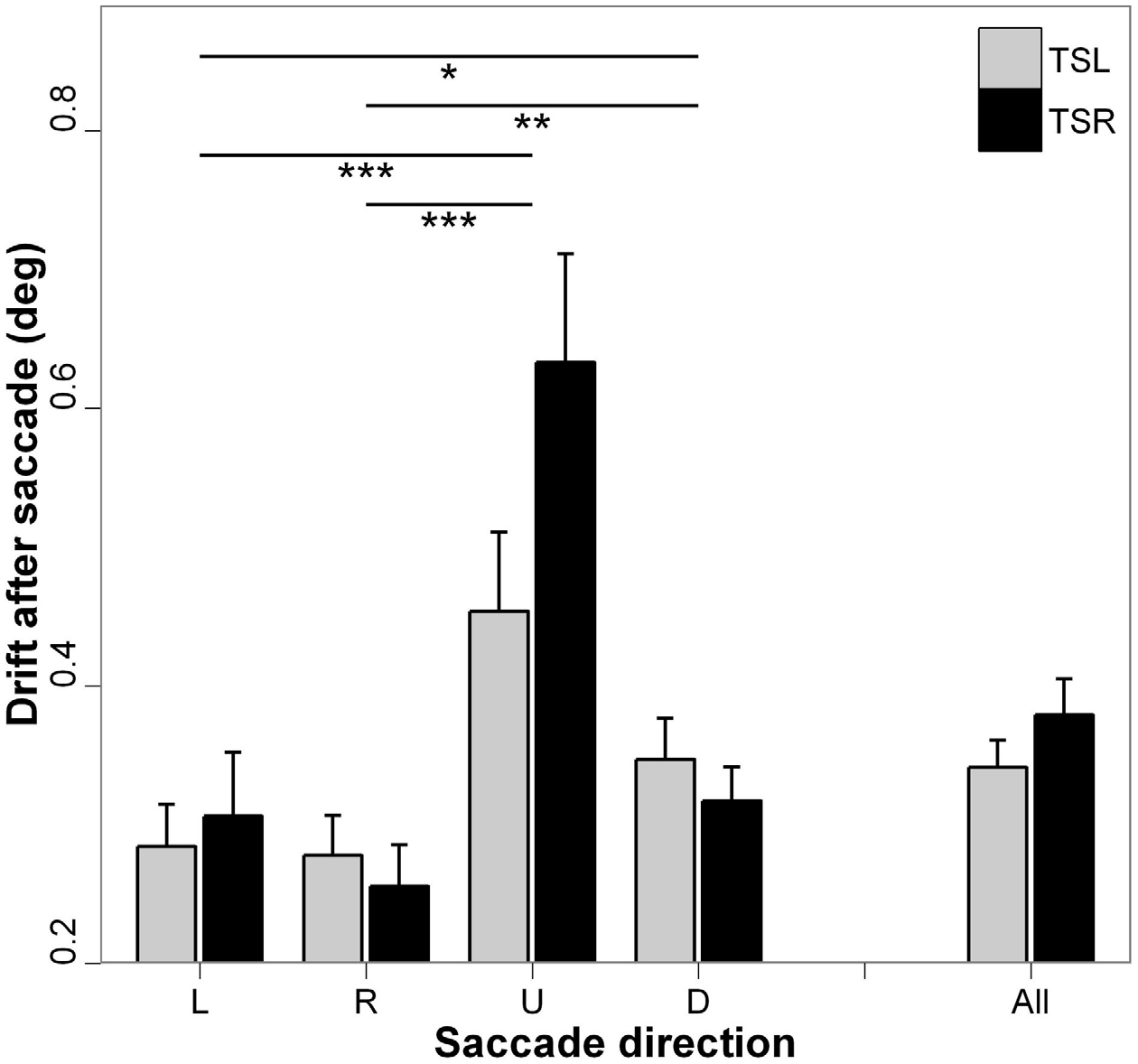
**Absolute mean values of fixation drift after saccades for each saccade direction (L, leftward; R, rightward; U, upward; D, downward) and tinnitus side (TSL, tinnitus side left; TSR, tinnitus side right).** Error bars represent the standard error. The symbol ^*^ indicates a statistically significant effect (^*^p < 0.05; ^**^p < 0.01; ^***^p < 0.001).

Figure 6 presents the absolute mean fixation drift after saccades and standard errors for each direction of the saccades and side of the tinnitus. ANOVA shows statistically significant main effect of the direction of the saccades [*F*_(3, 16)_ = 13.35; *p* < 0.001], i.e., vertical drift after vertical saccades is higher than horizontal drift after horizontal saccades (for each paired comparisons, *p* < 0.05; except for leftward vs. rightward saccades, *p* = 0.878, and upward vs. downward saccades, *p* = 0.277). There is no main effect of the tinnitus side [*F*_(1, 18)_ = 2.16; *p* = 0.142], and no interaction effect between tinnitus side and saccades direction [*F*_(3, 16)_ = 1.58; *p* = 0.192].

### Binocular coordination

**Figure 7:**
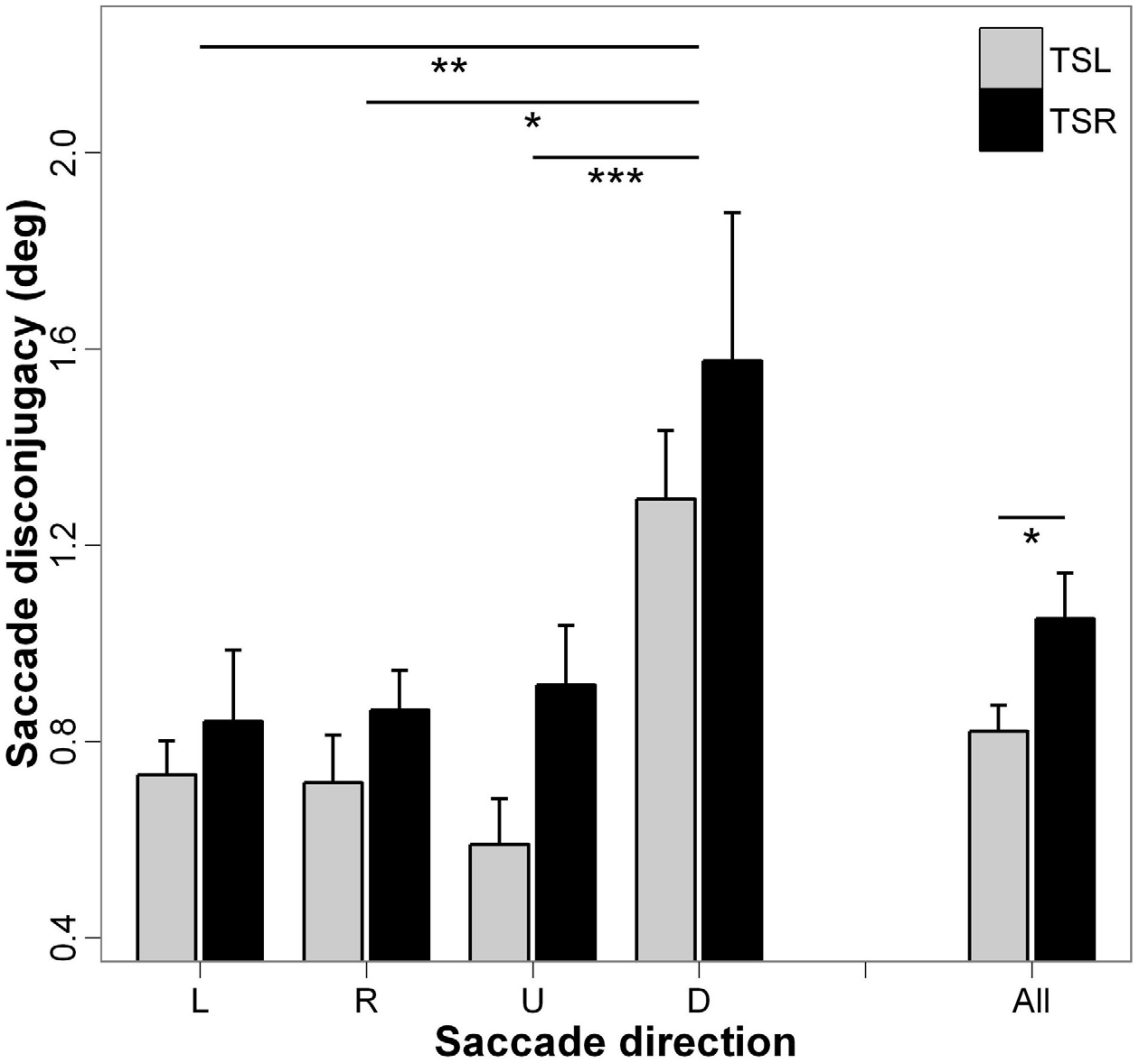
**Mean values of saccade disconjugacy for each saccade direction (L, leftward; R, rightward; U, upward; D, downward) and tinnitus side (TSL, tinnitus side left; TSR, tinnitus side right).** Error bars represent the standard error. The symbol ^*^ indicates a statistically significant effect (^*^p < 0.05; ^**^p < 0.01; ^***^p < 0.001).

Figure 7 presents the mean saccade disconjugacy and standard errors for each direction of the saccades and side of the tinnitus. ANOVA indicates statistically significant main effect of the tinnitus side [*F*_(1, 18)_ = 3.93; *p* < 0.05], i.e., subjects with right tinnitus presented higher disconjugated eye movements during saccades than those with left tinnitus, and a significant main effect of the direction of the saccades [*F*_(3, 16)_ = 5.72; *p* < 0.001], i.e., saccade disconjugacy is higher for downward saccades than that of upward (*p* < 0.001), leftward (*p* < 0.01), and rightward saccades (*p* < 0.05; others paired comparisons are n.s.). There is no interaction effect between tinnitus side and saccades direction [*F*_(3, 16)_ = 2.43; *p* = 0.064].

### Comparison with data from healthy adults

Table 2 summarizes the mean values of latency, of saccade amplitude and disconjugacy for leftward, rightward, upward, and downward saccades made by our tinnitus patients (TSL and TSR groups), and by 12 healthy subjects (mean age = 25 years old) in the studies of Vernet et al. (2008a,b). These latter subjects performed saccade movements in a similar paradigm as in the present study, i.e., interleaving 10° saccades toward a target presented in one of the four directions (left, right, up, down); the peripheral target was turned on after a 200-ms gap period (and not simultaneously as in the present experiment).

**Table 2:** **Mean values and standard deviation of latency, amplitude, and disconjugacy for leftward, rightward, upward, and downward saccades in control subjects (Vernet et al., 2008a,b) and tinnitus patients (TSL and TSR)**.

	**Control subjects**	**TSL**	**TSR**
**LATENCY (MS)**			
Leftward saccades	165 ± 31	168 ± 42 (*p* = 0.5056)	174 ± 48 (*p* = 0.0903)
Rightward saccades	166 ± 29	166 ± 39 (*p* = 1)	181 ± 56 (*p* = 0.0088)
Upward saccades	190 ± 26	184 ± 40 (*p* = 0.1696)	217 ± 67 (*p* = 0.0001)
Downward saccades	199 ± 30	201 ± 52 (*p* = 0.6967)	208 ± 49 (*p* = 0.0897)
**AMPLITUDE (DEG)**			
Leftward saccades	9.5 ± 0.5	8.8 ± 1.1 (*p* < 0.0001)	8.5 ± 1.9 (*p* < 0.0001)
Rightward saccades	9.5 ± 0.6	9.1 ± 1.4 (*p* = 0.0021)	8.8 ± 1.3 (*p* < 0.0001)
Upward saccades	9.4 ± 0.7	8.7 ± 1.8 (*p* = 0.0001)	7.9 ± 2.0 (*p* < 0.0001)
Downward saccades	9.4 ± 1.3	9.5 ± 1.8 (*p* = 0.6015)	10.0 ± 2.5 (*p* = 0.0189)
**DISCONJUGACY (DEG)**			
Leftward saccades	0.8 ± 0.4	0.7 ± 0.8 (*p* = 0.1894)	0.8 ± 1.3 (*p* = 1)
Rightward saccades	0.7 ± 0.4	0.7 ± 1.1 (*p* = 1)	0.9 ± 0.7 (*p* = 0.0070)
Upward saccades	0.6 ± 0.3	0.6 ± 1.0 (*p* = 1)	0.9 ± 1.1 (*p* = 0.0050)
Downward saccades	0.8 ± 0.4	1.3 ± 1.5 (*p* = 0.0002)	1.6 ± 2.7 (*p* = 0.0006)

p-values from t-test comparison with control data are given for both TSL and TSR.

In most cases, there are no significant differences between tinnitus patients and control subjects in terms of saccade latency; only rightward and upward saccades showed longer latencies in TSR patients than in controls. However, it has to be noted that the delay between the offset of the central target and the onset of the peripheral target (gap paradigm) is known to shorten the latency of saccades (Fisher, 1987). In addition, controls are younger than tinnitus patients and age is known to affect latency of saccades. Given this, it is difficult to jump to conclusions in terms of latency.

As far as we know, there is no evidence that age can affect saccade amplitude, at least for the two age ranges concerned. In particular, the study of Yang and Kapoula (2008) found that the amplitudes of vertical saccades were not significantly different in a group of young adults (20–28 years of age) and in a group of elderly adults (63–75 years of age). In the present set of data, amplitude hypometria appears to be significantly higher in patients compared with controls for leftward, rightward, and for upward saccades. For downward saccades, the amplitude was similar in TSL and controls but for TSR patients amplitudes were significantly larger than for control subjects. Downward saccades are known to be frequently hypermetric in controls (e.g., Collewijn et al., 1988b; Jagla et al., 1992) and this is perhaps magnified in tinnitus patients. Finally, saccade disconjugacy was significantly higher for TSR patients than for controls and for several directions (rightward, upward, and downward); for TSL patients, disconjugacy was higher than for controls only for downward saccades.

Overall, the data indicate abnormalities in the tinnitus patients in terms of saccade amplitude and disconjugacy that are more marked for right sided tinnitus.

## Discussion

The main findings are the following: spatial temporal analysis of eye movements highlights saccade differences in patients with predominantly right sided ST compared to those with predominantly left sided ST—i.e., latency, hypometria, and drift before saccade (along the saccade axis) are higher while velocity and quality of binocular coordination are lesser in TSR than in TSL patients. Most important, some of these effects are not general but specific to some directions. In particular, upward saccades presented increased latency and hypometria in TSR compared to TSL, the interaction effect between group (TSL/TSR) and saccade direction being significant in both cases. Moreover, horizontal saccades (leftward and rightward) were generally better performed than vertical saccades (downward and/or upward), regardless of the tinnitus side. Finally, relative to controls, amplitude, and disconjugacy of the saccades appear to be globally poorer in this tinnitus population compared with control subjects, specifically in TSR.

### Normality and abnormalities of saccades in tinnitus patients

Many recent studies reported eye movement abnormalities in tinnitus patients (e.g., Mezzalira et al., 2007; Kapoula et al., 2010; Yang et al., 2010). Kapoula et al. (2010) pointed out abnormalities in fixation, smooth pursuit, and optokinetic nystagmus in a few cases. In a more extensive study, they reported specific abnormalities in vergence eye movements, particularly in divergence, and abnormal interactions between saccade and vergence (Yang et al., 2010). The present study extends these findings as it shows abnormalities in terms of both saccade amplitude and disconjugacy, i.e., the quality of binocular coordination of saccades.

In line with these prior studies, the multiple eye movement abnormalities in tinnitus patients reflect dysfunction of many oculomotor subsystems (saccade, vergence, binocular coordination). Both cortical level, (e.g., parietal cortex), the brainstem as well as the cerebellum could be affected by such dysfunction. For instance, Vernet et al. (2008b) have shown that perturbation of the posterior parietal cortex (PPC) by transcranial magnetic stimulation (TMS) reduces binocular coordination of saccades. In addition, dysmetria of saccades and problems of binocular coordination can also result from cerebellum dysfunction (for review, see Leigh and Zee, 2006). As mentioned in the introduction, the neural circuitries at these different levels that are involved in eye movements supposedly interact with the neural plasticity induced by tinnitus.

### Directional asymmetries

The major effects found in this experiment indicate directional asymmetries that are specific to our tinnitus participants. First, horizontal saccades were globally better performed than vertical saccades, i.e., latencies and drifts prior and after saccades were all higher for both downward and upward saccades in comparison with rightward and leftward ones. Second, no left-right differences were found. Finally, these tinnitus patients showed specific vertical asymmetries of saccades: on the one hand, amplitudes were more hypometric and velocities lower for upward saccades in comparison with the other saccadic directions; on the other hand, binocular coordination was poorer for downward saccades as compared with other saccades.

#### Horizontal vs. vertical saccades

Horizontal and vertical saccades receive premotor commands from distinct burst neurons respectively located in the pontine and mesencephalic reticular formation (Leigh and Zee, 2006). Different patterns of event-related desynchronization (ERD) or event-related synchronization (ERS) have also been identified for vertical vs. horizontal eye movements (Kaiser et al., 2009). At the behavioral level, and in line with the reported results, eye movements from healthy subjects are known to be faster when saccades are made horizontally in comparison with vertical saccades, at least when they do not exceed 50° (Collewijn et al., 1988a,b). Directional asymmetries of latency, accuracy, and velocity of saccades have been investigated in several studies. However, most of them dealt either with horizontal (e.g., Weber and Fischer, 1995; Honda, 2002) or with vertical saccades (e.g., Heywood and Churcher, 1980; Honda and Findlay, 1992; Huaman and Sharpe, 1993; Schlykowa et al., 1996; Pitzalis and Di Russo, 2001; Zhou et al., 2002; Tzelepi et al., 2005, 2010) separately. In the following discussion, we will deal first with horizontal saccades and then with vertical saccades.

#### Horizontal saccades: no left-right asymmetry in tinnitus patients

The reported results do not indicate any left-right asymmetry neither for TSL nor for TSR. It has been argued that the horizontal rightward ocular motor system could be privileged due to scanning and reading habits (Manning et al., 1990; Chokron et al., 1998). However, a systematic left perceptual bias has been observed both in left-to-right and in right-to-left readers (Nicholls and Roberts, 2002). Left-right asymmetries as observed in perceptual judgment tasks are most probably due to an attentional bias induced by the dominance of the right hemisphere for spatial attention (Corbetta et al., 1995; Niemeier et al., 2008). Concerning experiments based on visually guided saccades, left-right asymmetry does not appear to systematically happen. Besides, some studies noticed that horizontal asymmetries of saccadic latencies could be idiosyncratic (Weber and Fischer, 1995; Honda, 2002).

#### Vertical saccades: marked upward deficit

The major effect found in the present study is the marked impairment of upward saccades in TSR group compared with saccades in the other directions in terms of almost all analyzed parameters (see Figures 2–6). This specific effect for upward saccades is also indicated by interaction effects which were significant only between upward vs. the other saccadic directions and the group (TSR vs. TSL) for both the latency and the amplitude. At the same time, binocular coordination was virtually the same in upward and horizontal saccades while it was poorer in downward saccades, TSR performing worse coordinated saccades than TSL.

Previous works already suggested that external physical sound can activate the vestibular system which can lead to body shift (Alessandrini et al., 2006). Hypothetically, phantom noise could trigger vestibular reactions similarly. And yet it is known that vestibular function is linked with different eye movements not limited to vestibuloocular reflex. In particular, cell activities in vestibular nuclei were found to be related to saccades (Chubb et al., 1984; Tomlinson and Robinson, 1984). Of interest, neurons discharging before vertical saccades and during vertical vestibular stimulation have also been identified in the interstitial nucleus of Cajal (Kaneko and Fukushima, 1998). It remains unclear whether tinnitus can affect the vestibular function in such a way that vertical saccades would be selectively defected. However, some diseases are known to affect specifically vertical saccades. For example, oculomotor deficit in progressive supranuclear palsy can start with impairment of either upward or downward saccades or both (Leigh and Zee, 2006). Niemann–Pick type C disease was also found to selectively affect vertical burst neurons (Rottach et al., 1997). Pending further investigations, we propose that tinnitus can induce subclinical vestibular dysfunction, affecting neurons implicated in the control of vertical saccades.

At the cortical level, TMS studies suggest that right and left parietal cortices are not identically involved in the control of vertical saccades. In particular, the right PPC has been found to be involved in the initiation of both downward and upward saccades (Tzelepi et al., 2005) while vertical saccades seem unaffected by TMS over the left PPC (Vernet et al., 2008c). In addition, Tzelepi et al. (2010) used magnetoencephalography and recorded higher frontal activity during the preparation of downward compared to upward saccades. Moreover, the superior parietal cortex is known to be associated with visuo-spatial attention activities (Corbetta et al., 1998). Many studies (e.g., Corbetta et al., 1998; Beauchamp et al., 2001; de Haan et al., 2008) indicate that saccadic and attentional processes are tightly integrated, at least at the neural level since anatomical overlap was demonstrated in frontal-parietal areas. In the context of cortical hemispheric asymmetries, the dominance of the right hemisphere has been evidenced for spatial attention both in lesions (e.g., Weintraub and Mesulam, 1987; Mosidze et al., 1994) and in non-brain damaged studies (e.g., Coull and Nobre, 1998; Nobre et al., 1999; see Mapstone et al., 2003) as well as for the initiation of saccades (e.g., Kapoula et al., 2004, 2005a,b; Yang and Kapoula, 2004).

We suggest that vertical asymmetries of saccades in tinnitus patients could be associated with maladaptive interactions between saccades, auditory, vestibular, and cortical circuitries. Auditory pathways will be discussed below.

### Auditory pathways asymmetry

In the context of tinnitus perception, the functional and structural asymmetries of auditory pathways have also to be underlined. Indeed there is a dominance of the right ear and audio-spatial attention has a prepotent rightward vector (Sosa et al., 2010) probably reflecting a left hemisphere predominance in auditory processing. Spontaneously, the right ear has a greater sensitivity for speech (McFadden and Mishra, 1993) and non-speech sounds (Todd et al., 2011) and displays more spontaneous and evoked acoustic emissions (Khalfa et al., 1998). Asymmetries in top-down medial olivo-cochlear (MOC) efferent system could account for such asymmetry leading to better integration of complex stimuli and thus to better speech to noise detection in the right auditory field (Giraud et al., 1997) even though some data indicate a stronger correlation between sound localization abilities and MOC functioning for the left ear (Andéol et al., 2011). The latter might be considered as a peripheral reflection of right-hemisphere dominance for spatial auditory processing in humans.

Those asymmetries could account for clinical discrepancies observed between left and right sided tinnitus. Indeed, left sided tinnitus is more frequent than right sided ones (Martines et al., 2010). Our short series is in line with these data displaying a prevalence of left sided tinnitus. Other data also suggest that left sided tinnitus are more intrusive (Mazurek et al., 2010). The tinnitus side also affects the correlation between tinnitus pitch and the frequency of maximum hearing loss, i.e., tinnitus pitch and hearing loss frequency are significantly correlated for right sided tinnitus and not for left sided ones (Schecklmann et al., 2012). On the other hand, it has been shown that only right sided tinnitus specifically impairs the classic right-ear advantage in a dichotic auditory listening task (Cuny et al., 2004a). Similarly, even if auditory attention is preferentially focused toward the tinnitus ear in case of unilateral tinnitus, tinnitus patients are better at categorizing a target sound in the right ear and/or to be less distracted by a deviant sound presented in the left ear (Cuny et al., 2004b).

Nevertheless, there is no consensus upon what is to be considered the actual neural correlate of tinnitus laterality. PET data suggest that left auditory cortices are always involved irrespective of tinnitus side (Langguth et al., 2006) whereas MRI, MEG, and EEG indicate that tinnitus generators could be located in the contralateral cortices (Weisz et al., 2007; van der Loo et al., 2009; Lefaucheur et al., 2012). But, subcortical structures could also be involved either bilaterally (i.e., inferior colliculi) according to fMRI data (Melcher et al., 2009) or asymmetrically (i.e., toward the contralateral parahippocampus) according to EEG data (Vanneste et al., 2011b).

## Conclusion

This study is the first to investigate systematically properties of saccades in patients with unilateral somatic tinnitus modulated by movements. In line with a prior case study from our team, we report here saccade abnormalities that interestingly are more accentuated in TSR than in TSL. The fact that saccade impairment was more marked for upward saccades in patients with right sided tinnitus remains the concern of the supposition and further investigations are clearly needed. Indeed, recent literature suggests that tinnitus is associated with complex neuroplastic maladaptive changes at the cortical and subcortical levels and in auditory as well as in non-auditory networks. However, these complex changes remain poorly understood. This study suggests that tinnitus can interact with ocular motor cortical areas, auditory pathways and maybe the vestibular function, leading to dysfunction of vertical saccades. Relative to horizontal saccades, vertical saccades are subtended by complex patterns of innervation distributed on the all extra-ocular muscles, by bilateral cortical activation and are perhaps more fragile.

### Conflict of interest statement

The authors declare that the research was conducted in the absence of any commercial or financial relationships that could be construed as a potential conflict of interest.
